# Type 2 Diabetes, PUFAs, and Vitamin D: Their Relation to Inflammation

**DOI:** 10.1155/2014/860703

**Published:** 2014-02-24

**Authors:** Ana L. Guadarrama-López, Roxana Valdés-Ramos, Beatríz E. Martínez-Carrillo

**Affiliations:** Center for Research and Graduate Studies in Health Sciences, Faculty of Medicine, Autonomous University of the State of Mexico, Paseo Tollocan Esquina, Jesús Carranza, Col. Moderna de la Cruz Toluca, 50180 México, MEX, Mexico

## Abstract

Chronic diseases have become one of the most important public health problems, due to their high costs for treatment and prevention. Until now, researchers have considered that the etiology of Type 2 diabetes mellitus (T2DM) is multifactorial. Recently, the study of the innate immune system has offered an explanation model of the pathogenesis of T2DM. On the other hand, there is evidence about the beneficial effect of polyunsaturated fatty acids (PUFA) n-3 and n-6 in patients with chronic inflammatory diseases including diabetes. Furthermore, high vitamin D plasmatic concentrations have been associated with the best performance of pancreatic **β** cells and the improving of this disease. In conclusion, certain fatty acids in the adequate proportion as well as 25-hydroxivitamin D can modulate the inflammatory response in diabetic people, modifying the evolution of this disease.

## 1. Introduction

Changes in human behavior and lifestyles in the last century have caused a great increase in prevalence of Type 2 diabetes mellitus. WHO calculates that 171 million people around the world have diabetes and that by 2030, its prevalence will reach epidemic proportions, affecting 366 million [1]. T2DM is considered a multifactorial disease, several hypotheses have tried to explain the origin of the pathology; that is, that it is an abnormality of the anterior hypothalamus and the endocrine pancreas caused by progressive ischemia or that there is abnormal islet innervation. Recently, there is increasing evidence that the acute phase inflammatory response induced by cytokines is closely related to the generation of insulin resistance and Type 2 diabetes mellitus. Some researchers have associated these pathologies with the presence of inflammatory and immune system biomarkers, including TNF-*α*, IL-1, IL-6, C Reactive Protein (CRP), monocyte chemotactic protein-1 (MCP-1), sialic acid, leptin, adiponectin, resistin, and visfatin [2].

Furthermore, recent epidemiological studies have associated total fat intake (saturated, mono, and poly-unsaturated fats) with T2DM; however, the type of fat could influence insulin metabolism positively, as can be observed when saturated fat is replaced with monounsaturated fat in the diet, improving considerably insulin action. There are several studies that demonstrate the beneficial effect of polyunsaturated fatty acid supplementation (n-3) in patients with active inflammatory processes [3], and others report that physiological concentrations of certain fatty acids can modulate the inflammatory response modifying the evolution of certain diseases [4].

Some epidemiological studies have recently reported an increase in serum 25-hydroxivitamin D (25-OH Vitamin D) deficiency in various populations around the world [5–9]. Until a few years ago, this deficiency had only been observed in very specific groups, such as old-age residents of high latitudes and altitudes or in low sun exposure. However, vitamin D deficiency has increasingly been found in apparently healthy populations as well as in industrialized society older adults [10].

The etiology of this deficiency has been considered multifactorial and ethnicity is one of these factors, in this sense, hypovitaminosis D has been observed in Afro-American and Latino populations who have a higher risk of insulin resistance and T2DM compared to Caucasian subjects [11]. Additionally, some researchers [12, 13] have hypothesized that low 25-OH vitamin D concentrations may have an important role in the pathogenesis of T2DM, although the mechanisms are not yet clear.

## 2. Type 2 Diabetes Mellitus

### 2.1. Genetic Factors

Positive family history confers a risk 2 to 4 times higher for T2DM. Fifteen to twenty-five percent of first-degree relatives of patients with T2DM have glucose intolerance or diabetes. Long-life risk (at age 80) for T2DM is 38% if one parent is affected and 60% at age 60 if both parents have the disease [14]. Although genetic factors are important, it is necessary to take into account that Diabetes is a multifactorial and heterogeneous disease [15].

### 2.2. Physiopathology of Hyperglycemia, Insulin Resistance, and Beta Pancreatic Cell Dysfunction

Insulin is the key hormone for glucose regulation and in general normoglycemia is maintained by the balance between secretion and action of insulin (less action, higher secretion and vice versa) and normal beta pancreatic cells can adapt to changes in insulin action [16].

### 2.3. Glucose Homeostasis

After a night of fasting, most of the glucose uptake occurs in tissues that are independent of insulin action, mainly in the nervous system and the splanchnic organs; this balance between uptake and glucose consumption is altered after feeding, when glucose homeostasis depends on three processes that normally occur as follows:

1. insulin secretion,
2. glucose uptake by peripheral tissues (85% in muscle and 4-5% in adipose tissue) and by splanchnic tissues (liver and intestine),
3. suppression of hepatic glucose production, which represents 85% of endogenous synthesis (the resting 15% is produced by the kidney) [17].

Although glucose uptake by adipose tissue is minimal, it is very important for maintaining glucose homeostasis as it coordinates fatty acid release from triacylglycerols, and produces cytokines that regulate insulin sensitivity in the liver and muscle. Alteration of any of these factors will produce glucose intolerance and open hyperglycemia. T2DM is characterized by the coexistence of two abnormalities:

- insufficiency in insulin secretion by pancreatic *β*-cells,
- insulin resistance [18].

### 2.4. Insulin Resistance

Insulin resistance is a decrease in the ability of insulin to exert its biological action at different glucose concentrations. Unless subjects with insulin resistance produce great amounts of insulin to compensate for its effects, they will surely develop hyperglycemia and diabetes. The defects related to insulin resistance include decreased expression of insulin receptors in the surface of those cells that are sensitive to this hormone which is due to alterations in the signaling pathways that should activate after insulin binding with its receptor and abnormalities in normal pathways that usually respond to insulin action such as glucose transportation and glucagon synthesis [19].

By the time blood glucose reaches the level of diagnosis for T2DM (≥126 mg/dL) the *β*-cell function disorders have already taken place. The inability of these to continue hypersecreting insulin is responsible for the transition from insulin resistance and compensating hyperinsulinism with normoglycemia to insulin resistance with noncompensating hyperinsulinism and glucose intolerance; ending up in insulin resistance with hyperinsulinemia and hyperglycemia [20].

### 2.5. Causes of Abnormalities in Insulin Secretion

#### 2.5.1. Alterations in *β* Cell Volume

*β*-cell volume is controlled by four independent mechanisms as follows:

1. mitosis of existing *β*-cells,
2. size of *β*-cells,
3. neogenesis from pancreatic epithelial cells,
4. *β*-cell apoptosis.

In normal conditions, approximately 0.5% of *β*-cells suffer apoptosis in adults, which is mainly compensated by mitosis and neogenesis, allowing an equilibrium between insulin production and metabolic needs [21].

There is evidence supporting the concept that *β*-cell apoptosis is an important factor in the decrease of islet number and the pathogenesis of T2DM [22]. Other related factors include stress of the endoplasmic reticulum, chronic hyperglycemia, oxidative stress, and the activity of some cytokines [23].

#### 2.5.2. Glucotoxicity

Hyperglycemia alone is capable of producing alterations in insulin secretion that decrease when it is corrected, that means that any increase in glycaemia in patients with a decrease in *β*-cell volume can cause important abnormalities in insulin secretion in the rest of the pancreatic tissue; it also contributes to the increase of insulin resistance and the defects in insulin secretion, which are corrected when glucose levels are reduced [24, 25].

#### 2.5.3. Lipotoxicity

Lipotoxicity is another cause of *β*-cell dysfunction. In normal acute conditions, *β*-cell exposure to physiological concentrations of free fatty acids stimulates insulin secretion; these are transformed into acyl coenzyme A (acyl-CoA) and subsequently into phosphatidic acid and diacylglycerol within *β*-cells [26]. These compounds activate specific isoforms of protein kinase C that stimulates insulin secretion. By comparison, chronic exposure to high concentrations of acyl-CoA inhibits insulin production through Randle's cycle. The increase in Acyl-CoA concentrations within *β*-cells also stimulates nitric oxide production, which increases inflammatory cytokine production, including IL-1 and TNF, that contributes to increase in the *β*-cell deterioration and apoptosis [25, 27].

#### 2.5.4. Defects in Function or Synthesis of Incretins

Oral intake of glucose stimulates insulin secretion, representing 50 to 70% of the normal insulin response; it depends on the entero-insular axis represented by two intestinal hormones that stimulate insulin secretion, called incretins: glucagon-like peptide-1 (GLP-1) and insulintrophic peptide dependent on glucose (GIP). These two hormones are liberated by endocrine cells in the duodenum and jejunum in response to carbohydrates in the intestine [28].

It has been observed that the action of these incretins is deteriorated or nonexistent in Type 2 diabetic patients, and it is actually considered one of the main mechanisms affecting insulin secretion in these patients [29].

### 2.6. Diagnosis

For the diagnosis of T2DM, it is convenient to use the current criteria of the American Diabetes Association (ADA) which are

- glycosylated hemoglobin ≥ 6.5% or fasting plasma glucose ≥ 126 mg/dL (7.0 mmol/L). Fasting is defined as noncaloric intake for at least 8 hours;
- plasma glucose at 2 hours ≥ 200 mg/dL (11.1 mmol/L) during a test of glucose tolerance;
- in patients with classic symptoms of hyperglycemia or hyperglycemic crises, a random plasma glucose ≥200 mg/dL (11.1 mmol/L) [20].

The process that determines the appearance of the disease is slow so that early detection is desirable; therefore, detection of cases with insulin resistance is a potential strategy that can facilitate the early diagnosis of this disease. However, insulin resistance alone denotes induction of glucose uptake in most tissues, but the relationship between secretion and action is a highly complex problem. In the presence of a severe insulin resistance, a disproportionate amount is secreted with a high percentage of immature forms of insulin being released into circulation, with a different half-life and action. Therefore a method to distinguish the immature forms of insulin is required.

There are useful methods for measuring glucose utilization by insulin as “the hyperinsulinemic euglycemic clamp” or the homeostasis model (HOMA-IR) [21]. The latter estimates severity of resistance more than insulin sensitivity, since the relationship between glucose and insulin at baseline reflects the balance between hepatic glucose production and secretion of insulin which is kept by feedback between the liver and pancreatic *β* cells and is obtained by dividing the product of glucose and insulin between 22.5 (when SI units are used) or between 405 (when expressed in mg/dL). The simplified formula is the result of a mathematical model which fits the action of insulin to the blood glucose value [22]. The cutoff point for diagnosis of insulin resistance may vary depending on the study population but is considered from 75th percentile of the study population. However there is data in Mexican population, placing HOMA in 2.4, with an additional advantage, since, in addition to insulin resistance, it allows to value beta cell function (HOMA-B), this value is obtained by dividing the product of insulin by 20 between at least 3.5 glucose, the cutoff point is also considered from the 75 percentile of the study population [23, 24].

## 3. Diabetes Mellitus and the Immune System

For more than 15 years, evidence has been gathered that supports the hypothesis that chronic low grade inflammation is a risk factor for the development of T2DM [15, 30–33]; however, the mechanisms are not clear yet. Existing theories include production of proinflammatory cytokines, such as IL-1 and TNF*α*, and increase in central fat mass, due to chronic inflammation [34].

Additionally acute phase proteins and certain cytokines are related with and through a great number of metabolic pathways that regulate insulin, the functions of lipoproteins lipases, and adipocytes, contributing to the development of insulin resistance [35].

Dietary factors may also increase acute phase proteins; for instance, a hyperenergetic diet increases protein C reactive concentrations, while a high fat diet increases sialic acid, which is considered a marker of the acute phase response and a cardiovascular and diabetes risk factor [36, 37].

In relation to the innate cellular immune system, macrophages have been associated with Type 2 diabetes mellitus pathogenesis. Proinflammatory M1 macrophages induce an inflammatory state and insulin resistance through inhibition of insulin signaling caused by IL-6 and TNF-alpha. Eguchi et al. [38] demonstrated a direct contribution of macrophages to beta cell dysfunction. Proinflammatory M1 macrophages were recruited to islets in mice infused with ethyl palmitate, in the *db/db* mouse, and in the KKAy mouse. Macrophage depletion *in vivo* in all these models increased Ins and Pdx1 mRNA expression in islets and increased glucose-stimulated insulin secretion *in vivo* and in isolated islets *ex vivo*.

Other cell populations affected are dendritic cells, it has been reported that hyperglycemic states determine a decrease of the total population of these, including myeloid dendritic cells type 1 (mDC1) and plasmacytoid dendritic cells (pDC) [39].

Natural Killer (NK) lymphocytes, which are important effector cells of the innate immune system, are responsible for controlling infections, but oxidative stress and endoplasmic reticulum (ER) stress induced by high glucose levels may influence NK cell function in T2D patients [40]. Some results demonstrate defects in NK cell-activating receptors NKG2D and NKp46 in T2D patients, and implicate the Unfolded Protein Response (UPR) pathway as a potential mechanism [41].

In relation to the adaptive immune system, there have been changes in certain cell lines, including a decreased polymorphonuclear leukocyte (PMNL) function; this alteration has been associated with defective chemotaxis, bacterial killing, leukotriene (LT) release, and lysosomal-enzyme secretion [42]. It has also been shown to produce increased levels of reactive oxygen species, possibly as a result of the effects of hyperglycemia [43]. Some studies have shown a correlation between impaired PMNL function and its improvement with adequate glycaemic control [44, 45].

T cells play an important role in the development of inflammatory processes, some experimental models suggest the association of an early T-lymphocyte occurrence in adipose tissue and the parallel initiation of insulin resistance (IR) in diet-induced obesity as a potential pathophysiological role of this cell type in the development of IR and T2DM [46, 47].

T reg cells are a subpopulation of CD4+ T cells that actively suppress physiologic and pathologic immune responses, therefore; contributing to the maintenance of immunological self-tolerance and immune homeostasis; it has been reported that T reg cells correlate with insulin resistance and glucose concentrations in T2DM patients [48].

## 4. Fatty Acids

Fats are organic biomolecules formed basically of carbon and hydrogen, and in a lesser extent of oxygen; they can be divided in phospholipids and triacylglycerols, both of which are made of fatty acids [49]. Fatty acids are long-chain monocarboxylic acids, with a pair number of carbon atoms, between 8 and 22. Fatty acids can be saturated, monounsaturated, or polyunsaturated according to the number of double bonds in the chain [50].

There are polyunsaturated fatty acids (PUFA) that the human organism cannot synthesize, such as linoleic acid (LA) and alpha linolenic acid (ALA) that must be obtained from the diet; they are called essential fatty acids (EFA). They belong to the n-6 or n-3 families of fatty acids, also known as *ω*-6 or *ω*-3, respectively [51]. EFAs can be converted into long chain fatty acids (LCFA) in the organism, with more double bonds such as arachidonic acid (AA), eicosapentaenoic acid (EPA), and docosahexaenoic (DHA) [9].

### 4.1. Polyunsaturated Fatty Acids and the Immune System

Animal or tissue culture studies as well as in human beings indicate that the amount and degree of saturation of fats in the diet influence inflammatory and immunologic responses. The nature of the effect depends on the type of fatty acid, age, health status, and experimental model [52–54].

Arachidonic acid and in a lesser proportion linoleic acid are the main components of the phospholipid membrane of lymphocytes, so growth and development of lymphoid tissues as well as the structural and functional integrity of lymphoid cells (T and B) are affected by essential fatty acid deficiency, which conditions a loss of functional integrity of T-CD4+ cells, monocytes, macrophages, and neutrophils, affecting chemotaxis and eicosanoid production [54].

*In vitro *experiments have shown that lymphocytes incorporate into their membranes a large amount of n-6 fatty acids (AA y LA) during their development and proliferation, which could lead to believe that their requirements are very high during the normal immune response in the secondary lymph nodes [54].

It is well known that activation of mature peripheral T-cells initiates with the interaction of the *T cell receptor* (TCR) and an antigenic peptide cleaved in a cell's Major Histocompatibility Complex (MHC). This interaction requires high concentrations of AA, and any change in this may affect TCR-MHC complex affinity and TCR signal transduction, determining the nature and magnitude of the T-cell response. There are *in vitro *studies showing the regulatory effect of AA metabolites on the development and function of immune system cells including growth and differentiation of thymocytes, proliferation and migration of T-cells, Th1 and Th2 response mediated by cytokines, antigen presentation, macrophage regulation, TNF*α*, IL-1 and IL-2 production, as well as suppressor T-cell induction [3, 53, 55–57].

Other *in vitro *studies have evaluated the effect of supplementing lymph node cell cultures with n-6 PUFA, showing that low concentrations of these improve B and T lymphocyte proliferation, while high concentrations inhibit them [58]. There is also evidence of reduction in proinflammatory cytokines IL-1 and TNF*α* production with gamma linoleic (GLA) and dihomogammalinoleic (DHLA) acids [53].

On the other hand, experimental research has given evidence on the effects of different n-3 PUFA on lymphocyte cultures. Additional data indicate that low concentrations of these PUFA stimulate lymphoproliferation of B and T cells, whereas high concentrations inhibit this effect [58, 59].

There are also some studies regarding the beneficial effects of n-3 PUFA in humans. Supplementation with n-3 rich fish oil has shown a decrease in helper T-cells [60], IL-1*β*, IL-2, TNF, IL-6, and IL-8 production [59, 61].

Existing scientific evidence suggests that moderate intake of the main n-6 and n-3 PUFA (arachidonic and linoleic acids), as well as an adequate proportion of both may be beneficial for those diseases that are related to the inflammatory process or immunity originated [52, 53, 57, 59, 62–65].

### 4.2. Polyunsaturated Fatty Acids and Type 2 Diabetes Mellitus

PUFAs may have a beneficial effect on the development or control of diabetes through several mechanisms. For instance, they are able to act as activators of peroxisome proliferator activated receptor gamma (PPAR*γ*); which stimulates the differentiation of preadipocytes to adipocytes, generating an increase in insulin receptors, thus reducing insulin resistance. Another mechanism is the protection of pancreatic beta cells from damage caused by an increase in free radicals produced in diabetes [66, 67].

Studies* in vitro *have reported that n-3 fatty acid supplementation improves the proinflammatory phenotype of macrophages, as well as insulin resistance in adipocytes [68].

Although there is strong evidence in humans and animal models that PUFAs exert a protective effect against the development of Type 2 diabetes mellitus [69–71], there are no concluding data in this respect. However, intake of diets rich in PUFA, particularly n-3 and n-6, has been shown to facilitate the action of insulin through various metabolic pathways, such as suppression of hepatic lipogenesis, reduction of the release of triacylglycerols from liver, improvement in ketogenesis, and oxidation of fatty acids in liver and skeletal muscle. All these mechanisms promote glucose uptake and decrease insulin resistance due to hypoactivity Δ5D desaturase and elevated activity of Δ6D and Δ9D desaturases [72].

Several investigations have been developed to evaluate the effect of fatty acid supplementation in the evolution of Type 2 diabetes mellitus [70, 71, 73–81]. Beneficial effects have been described on triacylglycerols, lipoproteins, haemostasia, atherogenic plaque stability, blood pressure, leukocyte function, glucose metabolism, insulin resistance, and even diabetic neuropathy; however, the results depend on dose and duration of intervention.

## 5. Vitamin D

Vitamin D or calciferol is an unsaponifiable heterolipid of the steroid group; it has two basic forms, D_2_ (ergocalciferol) found in plants as a product from ultraviolet B radiation on ergosterol and D_3_ which originates as dehydrocholesterol produced by ultraviolet B radiation, after becoming previtamin D_3_. Vitamin D_3_ can be synthesized in the human epidermis or ingested through fish oil, egg yolk, fortified foods, or supplements [82].

Vitamin D is converted into 25-hydroxivitamin D (25(OH)D) in the liver, which is the main circulating metabolite. Its measurement reflects intake and endogenous production; the active form is 1,25-dihydroxyvitamin D (1,25(OH)_2_D) or dihydroxycholecalciferol, which is hormone produced mainly in the kidney and regulated by parathyroid hormone, calcium, and phosphorous concentrations [83].

Vitamin D receptors are present in most tissues, including the endothelium, vascular smooth muscle, and myocardium; the first two are able to convert 25(OH)D into 1,25(OH)_2_D. Directly or indirectly 1,25(OH)_2_D has a role in the regulation of many genes, such as those involved in insulin production and development of vascular smooth muscle cells, which is the reason it is thought to be an important contributing factor to cardiovascular diseases [84].

### 5.1. Vitamin D and Type 2 Diabetes Mellitus

Epidemiologic data suggest that 9 out of 10 cases of T2DM can be attributed to modifiable lifestyles [85, 86]; however, changes in lifestyle are hard to accomplish and maintain in the long term. There is recent evidence in humans and animal models suggesting that vitamin D may play an important role in modifying the risk of diabetes [87].

Vitamin D receptors are present in pancreatic *β* and in immune system cells. Additionally, its role in the regulation of calcium absorption is well known; vitamin D participates in the activity of *β*-cell endopeptidases dependent on calcium and can act through two main pathways:

1. directly inducing *β*-cells to secrete insulin through an increase in intracellular calcium concentration through Ca channels,
2. by mediating *β*-cell calcium-dependent activation to facilitate conversion of proinsulin to insulin [88].

The role of vitamin D in the function of pancreatic cells can be mediated by the union of 1,25-dihydroxyvitamin D to its receptors in the beta cell. Alternatively, vitamin D can work through the activation of 25 hydroxyvitamin D (25(OH)D) by 1-alpha-hydroxylase expressed in pancreatic beta cells, directly improving insulin sensitivity by stimulating insulin receptor expression and the activation of PPAR-*δ* (peroxisome proliferator activated receptor delta), which has been associated with the regulation of fatty acid metabolism in skeletal muscle and adipose [89].

The expression of calbindin-D28K (vitamin D dependent on the union of proteins and calcium) has demonstrated a protective effect on beta cells from cytokine mediated cell death, reducing the risk of T2DM [88]. There are few studies in humans associating vitamin D and chronic inflammatory status of T2DM patients; however, the evidence suggests that vitamin D can improve insulin sensitivity and promote pancreatic *β*-cell survival by modulating the effects of cytokines and nuclear transcription factors such as NF-*κ*B [90].

Some cohort studies in the US and Finland have reported an association between vitamin D status and the risk of T2DM [91–93]. Some other studies [94, 95] have found associations between serum vitamin D levels, insulin resistance, and *β*-cell dysfunction.

Additionally, clinical studies have examined the effect of vitamin D supplementation and related indicators in different T2DM populations. These studies were carried out from 2 months to 7 years, while vitamin D doses were between 400 and 100,000 IU/day; some have reported improvements in central glycaemia [96], insulin sensitivity, and even lipid profile and endothelial function [97]. Other studies [98–102] of vitamin D supplementation without calcium showed no effect on glycaemia neither reduction in diabetes incidence after years of follow-up [103].

On the other hand, it has been demonstrated that vitamin D is a predictive factor for death by cardiovascular disease in T2DM patients [104, 105].

## 6. Adipokines

Adipose tissue has its own innervation and vascularization, with two morphological and functional distinct types of cells. White adipose tissue is dedicated to energy storage, while brown adipose tissue dissipates it [106]. Triacylglycerol and fatty acid storage in white adipose tissue are produced through the ability of insulin to stimulate glucose uptake and lipogenesis [107, 108].

It is well known that the distribution of energy within adipocytes due to an increase in adipose tissue mass or the amount of free circulating fatty acids, leads to obesity, dyslipidaemia, insulin resistance, and T2DM [109]; on the other hand, there is evidence indicating that the loss of adipose tissue in lipodystrophic syndromes results in insulin resistance and T2DM. These functions that are apparently opposite, can be explained by the functional complexity of adipose tissue and the great number of signaling molecules that it secretes, which are called “adipocytokines” or “adipokines;” however, not all of these peptides have cytokine family characteristics; therefore, the term “adipokine” is used for a protein produced and secreted only by adipocytes and not by other cells present in adipose tissue [110]. These polypeptides have been associated with various physiological processes such as food intake, energy balance, insulin action, glucose metabolism, vascular remodeling, and blood pressure regulation and coagulation [111, 112]; additionally, high levels of acute phase proteins and inflammatory cytokines in obese individuals have demonstrated that they suffer from a chronic state of low grade inflammation, which has been associated with the development of insulin resistance, metabolic syndrome, and T2DM [113, 114].

## 7. Leptin

Leptin is a 16 kDa hormone produced mainly by adipose tissue, and at a lesser extent by other tissues like muscle, stomach, and placenta [115, 116]; it also acts as a cytokine. Adipocytes secrete leptin in direct proportion to adipose tissue mass and nutritional status, being higher in subcutaneous in relation to visceral adipose tissue [117].

As a hormone, leptin helps monitor body weight (mainly by body fat content) to adjust the metabolic level, while as a cytokine, it can exert an effect on the innate and adaptive immune systems; leptin receptors have been found in neutrophils, monocytes, and lymphocytes. Leptin is able to activate proinflammatory cells, promoting a Th1 response and mediating TNF*α*, IL-2, or IL-6 production [118].

The effect of leptin on the immune system is explained by the fact that there are leptin receptors not only at the hypothalamus and adipose tissue, but also on cells of the immune system such as lymphocytes and monocytes [119]. Structurally, leptin receptors (Ob-R) belong to the class I cytokine family of receptors which include receptors for IL-2, IL-3, IL-4, IL-6, IL-7, and granulocyte-monocyte colony stimulating factor (GM-CSF) [118].

It is well known that leptin can affect T lymphocytes as they express ObR and high concentrations of leptin which seem to be associated with the production of proinflammatory cytokines by these cells [120, 121]. Additionally, leptin also acts on other immune cells, as has been demonstrated in human blood mononuclear cell cultures in presence of various amounts of leptin. The results of this study showed that leptin is able to induce dose-dependent mononuclear cell proliferation; to increase the expression of monocyte activation markers such as CD38, CD25, and CD71; and to increase TNF-*α* and IL-6 by cultured monocytes. The authors concluded that leptin could amplify monocyte activation and increase the proinflammatory response through cytokine production [122, 123].

Human macrophages and neutrophils also express a great amount of leptin receptors, causing chemotactic and apoptosis retardation. Additionally, dendritic cells may be playing a role on their development and function [118].

It has also been postulated that leptin may activate endothelial cells and stimulate macrophage activation in white adipose tissue (WAT). An increase in WAT and the consequent expression of inflammatory adipokines and decrease in adiponectin contribute to the chronic inflammatory state associated with obesity and the metabolic syndrome. Leptin has also been found to be involved in inflammation associated with atherosclerosis, acting as a signal for insulin sensitivity regulation in the organism. Leptin resistance has been identified as a causal factor of cardiovascular complications in obesity [124].

## 8. Adiponectin

Adiponectin (AMP1) is a protein hormone of 247 aminoacids, which is largely produced by WAT, it circulates in plasma at higher concentrations than the majority of hormones, its concentration is 5 to 30 *μ*g/mL, representing approximately 0.1% of all plasma proteins and in different amounts between genders, probably due to its regulation by sex hormones [125].

Adiponectin is an anti-inflammatory adipokine, showing improvement in hepatic insulin sensitivity, exerting a synergic effect with leptin, decreasing free nonesterified fatty acid flow, increasing fat oxidation, and reducing hepatic glucose release, plus stimulating glucose use by muscle [114, 126].

The effect of adiponectin on insulin sensitivity is mediated by an increase in oxidation of fatty acids through activated adenosine-monophosphate protein-kinase (AMPK) in skeletal muscle and liver, decreasing glucose synthesis.

As opposed to the majority of adipokines, adiponectin expression and its circulating concentrations are decreased in pathologies with insulin resistance and obesity. It has been demonstrated that Pima Indians have an inverse correlation between adiponectin concentrations and BMI, and that individuals with high adiponectin concentrations are less prone to develop T2DM in comparison to those with low concentrations [110, 114].

TNF-*α* and IL-6 are potent inhibitors of adiponectin expression and secretion in WAT biopsies and culture cells, suggesting that insulin resistance induction by TNF-*α* and IL-6 may also be caused by an inhibition of the autocrine-paracrine liberation of adiponectin [127]. It has been demonstrated that the administration of recombinant adiponectin in its complete or isolated form, exerts hypoglycemic effects, decreasing insulin resistance in murine models for obesity and diabetes [128].

In contrast, in lipoatrophic mice, insulin resistance was totally reversed with a combination of leptin and adiponectin at physiological doses, whereas the reversion was only partial when they were administered separately, showing that their joint effect can produce insulin sensitization in peripheral tissues [129].

## 9. Resistin

Resistin is a dimeric protein named for its apparent effect on the induction of insulin resistance in mice. It belongs to a family of cysteine-rich proteins called FIZZ (*found in inflammatory zone*), which were initially called Resistin-Like Molecules (RELM), and it has been found in adipocytes, macrophages, and other types of cells [130].

High plasma resistin concentrations have been found in experimental obesity models [131]; however, visceral adipose tissue has been associated with low concentrations of this adipokine [132]. In humans, resistin comes mainly from cells of the immune system and not from adipocytes. The possible effect of resistin on the development of insulin resistance has been evaluated; however, the results are not clear yet [133].

Experimental mice models have shown that recombinant resistin promotes insulin resistance and decreases insulin-stimulated glucose transporters in adipose tissue, while antiresistin antibodies produce the opposite effect [110, 128]. Furthermore, in humans, resistin may be implicated in inflammatory processes as mononuclear cells secrete it in important amounts. Resistin, IL-6, and TNF seem to influence each others action in monocytes through NF-kappa B specialized signaling routes [134].

## 10. Visfatin

Visfatin is another adipokine, initially thought to mimic insulin, although this is still in debate. It was first found in liver, skeletal muscle, and bone marrow, as a B-lymphocyte precursor growth factor, and was originally called pre-B cell colony stimulator. Its circulating values are related to WAT accumulation [135]. It is also produced by neutrophils preventing apoptosis by a mechanism that is mediated by caspases 3 and 8. It has been found to be elevated in patients with gastrointestinal inflammatory diseases [136]. There is also evidence that it induces chemotaxis, IL-1*β*, TNF, and IL-6 production, as well as synthesis of CD14+ monocyte costimulator molecules, increasing their ability to induce alloproliferative responses; it may also be involved in the regulation of the inflammatory response and other compensatory mechanisms [137].

The effects of visfatin are similar to insulin's, as it stimulates glucose transportation in muscle and adipocytes, and inhibits hepatic glucose production. Visfatin is forced to activate insulin receptors causing their phosphorylation and the activation of their signaling molecules; however, insulin and visfatin do not compete for the union to the insulin receptor, indicating that they are recognized by different regions [138].

In a KKAy mouse model for obesity and diabetes mellitus, the expression of visfatin in visceral fat tissue and its serum concentrations was found to be directly associated with the increase in obesity in animals fed a high fat diet compared to controls [139].

## 11. Cytokines

Cytokines are a large group of low molecular weight proteins that mainly regulate the immune response; however, they may have other functions such as embryogenesis, cellular differentiation, and migration, amongst others. They are mainly produced by leukocytes, but some of them can also be secreted by other cells. They were originally called “lymphokines” as they were considered biological products of lymphocytes in response to antigens [140].

In general, these molecules are not constitutively produced, cell activation is necessary for them to be produced in sufficient quantities to exert a biological effect. Most cytokines are secreted in a glycosylated form which increases their stability and solubility [141]. Cytokines have a very short half-life and act at very low concentrations through the union with high affinity receptors. They exert an autocrine effect when anchored to receptors in the producing cell; they also have paracrine effects on other adjoining cells, while in some cases they can be released into the blood or lymphatic circulation, exerting their effect on other organs and tissues acting as hormones [142].

Regarding the inflammatory response, some cytokines promote its development (proinflammatory), while others suppress it (anti-inflammatory) [143].

## 12. Tumor Necrosis Factor Alpha (TNF-*α*)

TNF-*α* was the first cytokine associated with insulin resistance in animals; it is overexpressed in adipose tissue in obesity and decreases with weight loss, being also considered as an important insulin resistance regulator [144]. It has been found to affect insulin signaling *in vivo *and *in vitro*, decreasing the expression of adiponectin in adipose tissue [145]. It stimulates lipolysis, inhibiting the expression of lipoprotein lipase (LPL) and glucose transporter 4 (GLUT 4), which are two key elements for fat accumulation thus, it could be considered a mechanism for the reduction of large fatty acids. However, high concentrations of TNF-*α* could also be implied in the development of metabolic abnormalities such as insulin resistance [127].

TNF-*α* seems to play a role in the physiopathology of blood hypertension (BHP) associated with obesity and insulin resistance, as it inhibits insulin dependent glucose uptake by interfering with its signaling [30, 127].

## 13. Interleukin 1 (IL-1)

IL-1 is an obesity and insulin resistance associated cytokine, produced by several types of cells, but mainly by activated macrophages. It is a key mediator of the inflammatory response, with stimulator and inhibitor actions on some cells, even promoting apoptosis in others. It is able to promote the expression of the same genes that produce it, as well as synthesis of prostaglandins, leukotrienes, interleukin-8, and certain protooncogenes like *c-fos* y *c-jun *[146].

Insulin signaling is directly affected by IL-1 through the induction of cytokine-3 signaling suppressor (SOCS-3); it is mainly stimulated by TNF-*α* and catecholamines and inhibited by glucocorticoids. It has multiple effects on various tissues, acting together with IL-6 as an endogenous pyrogens, stimulating thermogenesis [147]. It is also a regulator of Protein C Reactive (PCR) hepatic production, and its increase in adipose tissue may stimulate PCR synthesis, which is another inflammatory response modulator [126, 148, 149].

In the past years, it has been demonstrated that high concentrations of glucose stimulate IL-1*β* production by *β*-cells [31], implying an effect on the development of T2DM. The evidence suggests the presence of an inflammatory process that leads to failure of the *β*-cell to secrete sufficient amounts of insulin in diabetic patients. Insulitis is associated with the pathologic activation of the innate inflammatory system by metabolic stress, which is mediated by IL-1 signaling causing lesions in the pancreatic parenchyma [32].

Overnutrition is the main cause of Type 2 diabetes. Exposure of pancreatic islets to glucose or free fatty acids induces production and release of IL-1*β* [33, 146, 150], which may also be induced by leptin [151]. IL-1*β* gene expression has been found to be 100 times increased in beta cells of islets from T2DM patients compared to nondiabetic controls [33].

Fatty acids from adipocytes, which are also a source of IL-1*β* [152], could amplify these signals by self-activation through their own IL-1 [33, 153].

## 14. Conclusions

**Figure 1:**
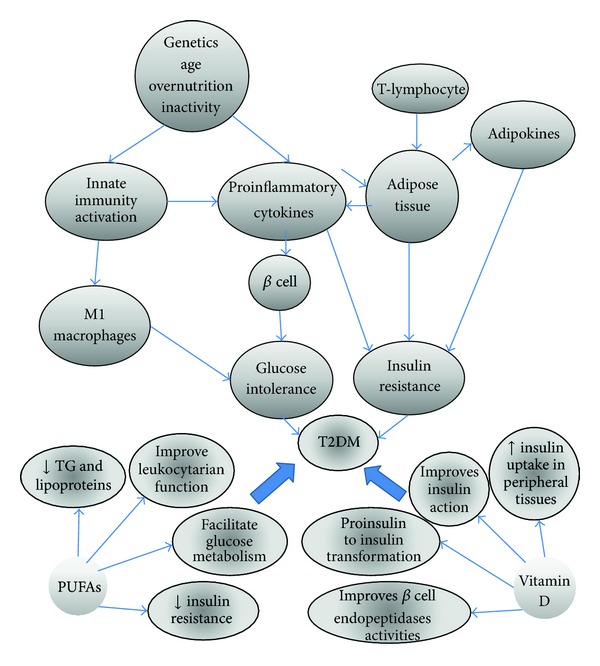
Diverse factors such as overnutrition, physical inactivity, age, and genetics can activate the innate immune system and consequently the production of cytokines, which by themselves lead to insulin resistance and diabetes, but there is evidence that some of these alterations can be improved through the consumption of PUFAs and vitamin D.

Recent epidemiological studies have associated total fat intake (saturated, mono, and poly-unsaturated fats) with T2DM; however, the type of fat could influence insulin metabolism positively, as can be observed when saturated fat is replaced with monounsaturated fat in the diet, improving considerably insulin action. There are several studies that demonstrate the beneficial effect of polyunsaturated fatty acid supplementation (n-3) in patients with active inflammatory processes and others that report that physiologic concentrations of certain fatty acids can modulate the inflammatory response modifying the evolution of some diseases including Type 2 diabetes. On the other hand, some epidemiological studies have recently reported an increase in serum 25-hydroxivitamin D (25-OH vitamin D) deficiency in diabetic populations. The etiology of this deficiency has been considered multifactorial and ethnicity is one of these factors; low 25-OH vitamin D concentrations may have an important role in the pathogenesis of T2DM, although the mechanisms are not yet clear. Some of these alterations are resumed in Figure 1.

## References

[1] WHO World Health Statics. http://www.who.int/gho/publications/world_health_statistics/EN_WHS2011_TOC.pdf.

[2] Bulló M, García-Lorda P, Salas-Salvadó J (2002). Plasma soluble tumor necrosis factor alpha receptors and leptin levels in normal-weight and obese women: effect of adiposity and diabetes. *European Journal of Endocrinology*.

[3] Calder PC (2003). n-3 polyunsaturated fatty acids and inflammation: from molecular biology to the clinic. *Lipids*.

[4] Chokkalingam K, Tsintzas K, Norton L, Jewell K, Macdonald IA, Mansell PI (2007). Exercise under hyperinsulinaemic conditions increases whole-body glucose disposal without affecting muscle glycogen utilisation in type 1 diabetes. *Diabetologia*.

[5] Holick MF (2005). Vitamin D deficiency in CKD: why should we care?. *American Journal of Kidney Diseases*.

[6] Holick MF (2005). Vitamin D: important for prevention of osteoporosis, cardiovascular heart disease, type 1 diabetes, autoimmune diseases, and some cancers. *Southern Medical Journal*.

[7] Moore CE, Murphy MM, Holick MF (2005). Vitamin D intakes by children and adults in the United States differ among ethnic groups. *Journal of Nutrition*.

[8] Grant WB, Holick MF (2005). Benefits and requirements of vitamin D for optimal health: a review. *Alternative Medicine Review*.

[9] Raiten DJ, Picciano MF (2004). Vitamin D and health in the 21st century: bone and beyond. Executive summary. *The American Journal of Clinical Nutrition*.

[10] Hidayat RS, Setiati S, Soewondo P (2010). The association between vitamin D deficiency and type 2 diabetes mellitus in elderly patients. *Acta medica Indonesiana*.

[11] Chiu KC, Chuang L-M, Yoon C (2001). The vitamin D receptor polymorphism in the translation initiation codon is a risk factor for insulin resistance in glucose tolerant Caucasians. *BMC Medical Genetics*.

[12] Bolland MJ, Chiu WW, Davidson JS (2008). The effects of seasonal variation of 25-hydroxyvitamin D on diagnosis of vitamin D insufficiency. *New Zealand Medical Journal*.

[13] Tzotzas T, Papadopoulou FG, Tziomalos K (2010). Rising serum 25-hydroxy-vitamin D levels after weight loss in obese women correlate with improvement in insulin resistance. *Journal of Clinical Endocrinology and Metabolism*.

[14] Heideman WHN, Nierkens V, Stronks K (2011). DiAlert: a lifestyle education programme aimed at people with a positive family history of type 2 diabetes and overweight, study protocol of a randomised controlled trial. *BMC Public Health*.

[15] Pickup JC, Crook MA (1998). Is type II diabetes mellitus a disease of the innate immune system?. *Diabetologia*.

[16] Kahn SE (2003). The relative contributions of insulin resistance and beta-cell dysfunction to the pathophysiology of Type 2 diabetes. *Diabetologia*.

[17] Stumvoll M, Goldstein BJ, Van Haeften TW (2005). Type 2 diabetes: principles of pathogenesis and therapy. *The Lancet*.

[18] Weyer C, Bogardus C, Mott DM, Pratley RE (1999). The natural history of insulin secretory dysfunction and insulin resistance in the pathogenesis of type 2 diabetes mellitus. *Journal of Clinical Investigation*.

[19] Mercurio V, Carlomagno G, Fazio V, Fazio S (2012). Insulin resistance: is it time for primary prevention?". *World Journal of Cardiology*.

[20] Tébar-Massó FJE-J (2009). *La Diabetes en la Práctica Clínica*.

[21] Van Haeften TW, Pimenta W, Mitrakou A (2002). Disturbances in *β*-cell function in impaired fasting glycemia. *Diabetes*.

[22] Butler AE, Janson J, Soeller WC, Butler PC (2003). Increased *β*-cell apoptosis prevents adaptive increase in *β*-cell mass in mouse model of type 2 diabetes: evidence for role of islet amyloid formation rather than direct action of amyloid. *Diabetes*.

[23] Laybutt DR, Preston AM, Åkerfeldt MC (2007). Endoplasmic reticulum stress contributes to beta cell apoptosis in type 2 diabetes. *Diabetologia*.

[24] Scheen AJ, Paquot N, Lefebvre PJ (1999). Glucoxocity and lipotoxicity, two partners in the vicious circle of type 2 diabetes. *Revue Medicale de Liege*.

[25] Sivitz WI (2001). Lipotoxicity and glucotoxicity in type 2 diabetes: effects on development and progression. *Postgraduate Medicine*.

[26] Kim JW, Yoon KH (2011). Glucolipotoxicity in Pancreatic beta-Cells. *Diabetes & Metabolism Journal*.

[27] Poitout V, Robertson RP (2002). Minireview: secondary *β*-cell failure in type 2 diabetes—a convergence of glucotoxicity and lipotoxicity. *Endocrinology*.

[28] Lotfy MS, Singh J, Kalász H, Tekes K, Adeghate E (2011). Medicinal chemistry and applications of incretins and DPP-4 inhibitors in the treatment of type 2 diabetes mellitus. *Open Medicinal Chemistry Journal*.

[29] Ishii H, Sato Y, Takei M, Nishio S, Komatsu M (2011). Glucose-incretin interaction revisited. *Endocrine Journal*.

[30] Fernández-Real JM (2003). El adipocito como Biomarcador. *Endocrinología y Nutrición*.

[31] Maedler K, Sergeev P, Ris F (2002). Glucose-induced *β* cell production of IL-1*β* contributes to glucotoxicity in human pancreatic islets. *Journal of Clinical Investigation*.

[32] Donath MY, Størling J, Berchtold LA, Billestrup N, Mandrup-Poulsen T (2008). Cytokines and *β*-cell biology: from concept to clinical translation. *Endocrine Reviews*.

[33] Böni-Schnetzler M, Thorne J, Parnaud G (2008). Increased interleukin (IL)-1*β* messenger ribonucleic acid expression in *β*-cells of individuals with type 2 diabetes and regulation of IL-1*β* in human islets by glucose and autostimulation. *Journal of Clinical Endocrinology and Metabolism*.

[34] Bays H, Mandarino L, DeFronzo RA (2004). Role of the adipocyte, free fatty acids, and ectopic fat in pathogenesis of type 2 diabetes mellitus: peroxisomal proliferator-activated receptor agonists provide a rational therapeutic approach. *Journal of Clinical Endocrinology and Metabolism*.

[35] Crook M (2004). Type 2 diabetes mellitus: a disease of the innate immune system? An update. *Diabetic Medicine*.

[36] Liu SJM, Manson JE, Buring JE, Stampfer MJ, Willett WC, Ridker PM (2002). Relation between a diet with a high glycemic load and plasma concentrations of high-sensitivity C-reactive protein in middle-aged women1-3. *American Journal of Clinical Nutrition*.

[37] Coppack SW (2001). Pro-inflammatory cytokines and adipose tissue. *Proceedings of the Nutrition Society*.

[38] Eguchi K, Manabe I, Oishi-Tanaka Y (2012). Saturated fatty acid and TLR signaling link *β* cell dysfunction and islet inflammation. *Cell Metabolism*.

[39] Seifarth CC, Hinkmann C, Hahn E-C, Lohmann T, Harsch IA (2008). Reduced frequency of peripheral dendritic cells in type 2 diabetes. *Experimental and Clinical Endocrinology and Diabetes*.

[40] Zhang K, Kaufman RJ (2008). Chapter twenty identification and characterization of endoplasmic reticulum stress-induced apoptosis in vivo. *Methods in Enzymology*.

[41] Berrou J, Fougeray S, Venot M (2013). Natural killer cell function, an important target for infection and tumor protection, is impaired in type 2 diabetes. *PLoS ONE*.

[42] Hopps E, Camera A, Caimi G (2008). Polimorphonuclear leukocytes and diabetes mellitus. *Minerva Medica*.

[43] Hand WL, Hand DL, Vasquez Y (2007). Increased polymorphonuclear leukocyte respiratory burst function in type 2 diabetes. *Diabetes Research and Clinical Practice*.

[44] Bhattacharya SK, Shastri S, Mahajan P (2007). Polymorphonuclear leukocyte function in type-2 diabetes mellitus patients and its correlation with glycaemic control. *Nepal Medical College Journal*.

[45] Rayaman PR, Çevikbaş A, Demirtunç R, Şehirli AÖ, Gürer ÜS (2013). The effect of some antibiotics on Polymorphonuclear Leukocyte (PMN) functions and PMN'S myeloperoxidase activity, glutathione and malondialdehyde levels of patients with type 2 diabetes mellitus in vitro. *Journal of Marmara University Institute of Health Sciences*.

[46] Kintscher U, Hartge M, Hess K (2008). T-lymphocyte infiltration in visceral adipose tissue: a primary event in adipose tissue inflammation and the development of obesity-mediated insulin resistance. *Arteriosclerosis, Thrombosis, and Vascular Biology*.

[47] Xu H, Barnes GT, Yang Q (2003). Chronic inflammation in fat plays a crucial role in the development of obesity-related insulin resistance. *Journal of Clinical Investigation*.

[48] Miyara M, Sakaguchi S (2007). Natural regulatory T cells: mechanisms of suppression. *Trends in Molecular Medicine*.

[49] Voet DV, Pratt C J (2009). *Fundamentos de Bioquímica*.

[50] Melo VCO (2000). *Bioquímica de los Procesos Metabólicos*.

[51] Sprecher H, Braceo UD, Deckelbaum RJ (1992). Long chain fatty acid metabolism. *Polyunsaturated Fatty Acids in Human Nutrition*.

[52] Vafeiadou KW, Weech M, Sharma V (2012). A review of the evidence for the effects of total dietary fat, saturated, monounsaturated and n-6 polyunsaturated fatty acids on vascular function, endothelial progenitor cells and microparticles. *British Journal of Nutrition*.

[53] Harbige LS (1998). Dietary n-6 and n-3 fatty acids in immunity and autoimmune disease. *Proceedings of the Nutrition Society*.

[54] Harbige LS (2003). Fatty acids, the immune response, and autoimmunity: a question of n-6 essentiality and the balance between n-6 and n-3. *Lipids*.

[55] Merzouk SA, Saker M, Reguig KB (2008). N-3 polyunsaturated fatty acids modulate in-vitro T cell function in type I diabetic patients. *Lipids*.

[56] Alnajjar A, Chabane Sari D, Abuharfeil N, Hudaib M, Aburjai T (2006). Effect of n-3 and n-6 polyunsaturated fatty acids on lymphocyte proliferation, interleukin production and phospholipid fatty acids composition in type 2 diabetic and healthy subjects in Jordan people. *Prostaglandins Leukotrienes and Essential Fatty Acids*.

[57] Calder PC (1998). Dietary fatty acids and the immune system. *Nutrition Reviews*.

[58] Calder PC (1998). Symposium on “Lipids and the immune system”. Dietary fatty acids and lymphocyte functions. *Proceedings of the Nutrition Society*.

[59] Calder PC (1998). Immunoregulatory and anti-inflammatory effects of n-3 polyunsaturated fatty acids. *Brazilian Journal of Medical and Biological Research*.

[60] Meydani SN, Lichtenstein AH, Cornwall S (1993). Immunologic effects of national cholesterol education panel step-2 diets with and without fish-derived n-3 fatty acid enrichment. *Journal of Clinical Investigation*.

[61] Endres S, Ghorbani R, Kelley VE (1989). The effect of dietary supplementation with n-3 polyunsaturated fatty acids on the synthesis of interleukin-1 and tumor necrosis factor by mononuclear cells. *The New England Journal of Medicine*.

[62] Calder PC (2003). New evidence in support of the cardiovascular benefit of long-chain n-3 fatty acids. *Italian Heart Journal*.

[63] Calder PC (2003). Long-chain n-3 fatty acids and inflammation: potential application in surgical and trauma patients. *Brazilian Journal of Medical and Biological Research*.

[64] Calder PC (1996). Effects of fatty acids and dietary lipids on cells of the immune system. *Proceedings of the Nutrition Society*.

[65] Yaqoob P, Calder PC (2003). n-3 polyunsaturated fatty acids and inflammation in the arterial wall. *European Journal of Medical Research*.

[66] Suresh Y, Das UN (2003). Long-chain polyunsaturated fatty acids and chemically induced diabetes mellitus: effect of *ω*-3 fatty acids. *Nutrition*.

[67] Suresh Y, Das UN (2003). Long-chain polyunsaturated fatty acids and chemically induced diabetes mellitus: effect of *ω*-6 fatty acids. *Nutrition*.

[68] Oliver EM, McGillicuddy FC, Harford KA (2011). Docosahexaenoic acid attenuates macrophage-induced inflammation and improves insulin sensitivity in adipocytes-specific differential effects between LC n-3 PUFA. *Journal of Nutritional Biochemistry*.

[69] Storlien LH, Kraegen EW, Chisholm DJ (1987). Fish oil prevents insulin resistance induced by high-fat feeding in rats. *Science*.

[70] Kabir M, Skurnik G, Naour N (2007). Treatment for 2 mo with n-3 polyunsaturated fatty acids reduces adiposity and some atherogenic factors but does not improve insulin sensitivity in women with type 2 diabetes: a randomized controlled study. *American Journal of Clinical Nutrition*.

[71] Mostad IL, Bjerve KS, Lydersen S, Grill V (2008). Effects of marine n-3 fatty acid supplementation on lipoprotein subclasses measured by nuclear magnetic resonance in subjects with type II diabetes. *European Journal of Clinical Nutrition*.

[72] Vessby B, Aro A, Skarfors E, Berglund L, Salminen I, Lithell H (1994). The risk to develop NIDDM is related to the fatty acid composition of the serum cholesterol esters. *Diabetes*.

[73] Shah M, Adams-Huet B, Brinkley L, Grundy SM, Garg A (2007). Lipid, glycemic, and insulin responses to meals rich in saturated, cis-monounsaturated, and polyunsaturated (n-3 and n-6) fatty acids in subjects with type 2 diabetes. *Diabetes Care*.

[74] Waite JLN, Hart K, Robertson D, Badley E, Burton S (2008). The impact of fish-oil supplements on insulin sensitivity. *Journal of Human Nutrition and Dietetics*.

[75] Barre DE, Mizier-Barre KA, Griscti O, Hafez K (2008). High dose flaxseed oil supplementation may affect fasting blood serum glucose management in human type 2 diabetics. *Journal of Oleo Science*.

[76] Garg ML, Blake RJ, Clayton E (2007). Consumption of an n-3 polyunsaturated fatty acid-enriched dip modulates plasma lipid profile in subjects with diabetes type II. *European Journal of Clinical Nutrition*.

[77] de Luis DA, Conde R, Aller R (2009). Effect of omega-3 fatty-acids on cardiovascular risk factors in patients with type 2 diabetes mellitus and hypertriglyceridemia: an open study. *European Review for Medical and Pharmacological Sciences*.

[78] Holman RR, Paul S, Farmer A, Tucker L, Stratton IM, Neil HAW (2009). Atorvastatin in factorial with omega-3 EE90 risk reduction in diabetes (AFORRD): a randomised controlled trial. *Diabetologia*.

[79] Nomura S, Shouzu A, Omoto S (2009). Effects of eicosapentaenoic acid on endothelial cell-derived microparticles, angiopoietins and adiponectin in patients with type 2 diabetes. *Journal of Atherosclerosis and Thrombosis*.

[80] Rizza S, Tesauro M, Cardillo C (2009). Fish oil supplementation improves endothelial function in normoglycemic offspring of patients with type 2 diabetes. *Atherosclerosis*.

[81] Stirban A, Nandrean S, Götting C (2010). Effects of n-3 fatty acids on macro- and microvascular function in subjects with type 2 diabetes mellitus. *American Journal of Clinical Nutrition*.

[82] Lopes N, Sousa B, Martins D (2010). Alterations in Vitamin D signalling and metabolic pathways in breast cancer progression: a study of VDR, CYP27B1 and CYP24A1 expression in benign and malignant breast lesions Vitamin D pathways unbalanced in breast lesions. *BMC Cancer*.

[83] Arnson Y, Amital H, Shoenfeld Y (2007). Vitamin D and autoimmunity: new aetiological and therapeutic considerations. *Annals of the Rheumatic Diseases*.

[84] Jones BJ, Twomey PJ (2008). Issues with vitamin D in routine clinical practice. *Rheumatology*.

[85] Hu FB, Manson JE, Stampfer MJ (2001). Diet, lifestyle, and the risk of type 2 diabetes mellitus in women. *The New England Journal of Medicine*.

[86] Lindström J, Ilanne-Parikka P, Peltonen M (2006). Sustained reduction in the incidence of type 2 diabetes by lifestyle intervention: follow-up of the Finnish Diabetes Prevention Study. *The Lancet*.

[87] Pittas AG, Lau J, Hu FB, Dawson-Hughes B (2007). Review: the role of vitamin D and calcium in type 2 diabetes. A systematic review and meta-analysis. *Journal of Clinical Endocrinology and Metabolism*.

[88] Mathieu C, Gysemans C, Giulietti A, Bouillon R (2005). Vitamin D and diabetes. *Diabetologia*.

[89] van Etten E, Mathieu C (2005). Immunoregulation by 1,25-dihydroxyvitamin D3: basic concepts. *Journal of Steroid Biochemistry and Molecular Biology*.

[90] Seshadri KGT, Rajendran A B (2011). Role of vitamin D on diabetes. *The Journal of Clinical Endocrinology and Metabolism*.

[91] Knekt P, Laaksonen M, Mattila C (2008). Serum vitamin D and subsequent occurrence of type 2 diabetes. *Epidemiology*.

[92] Pittas AG, Dawson-Hughes B, Li T (2006). Vitamin D and calcium intake in relation to type 2 diabetes in women. *Diabetes Care*.

[93] Liu S, Song Y, Ford ES, Manson JE, Buring JE, Ridker PM (2005). Dietary calcium, vitamin D, and the prevalence of metabolic syndrome in middle-aged and older U.S. Women. *Diabetes Care*.

[94] Kayaniyil S, Vieth R, Retnakaran R (2010). Association of vitamin D with insulin resistance and beta-cell dysfunction in subjects at risk for type 2 diabetes. *Diabetes Care*.

[95] Broder AR, Tobin JN, Putterman C (2010). Disease-specific definitions of vitamin D deficiency need to be established in autoimmune and non-autoimmune chronic diseases: a retrospective comparison of three chronic diseases. *Arthritis Research and Therapy*.

[96] Pittas AG, Harris SS, Stark PC, Dawson-Hughes B (2007). The effects of calcium and vitamin D supplementation on blood glucose and markers of inflammation in nondiabetic adults. *Diabetes Care*.

[97] Shab-Bidar S, Neyestani TR, Djazayery A (2011). Regular consumption of vitamin D-fortified yogurt drink (Doogh) improved endothelial biomarkers in subjects with type 2 diabetes: a randomized double-blind clinical trial. *BMC Medicine*.

[98] Nilas L, Christiansen C (1984). Treatment with vitamin D or its analogues does not change body weight or blood glucose level in postmenopausal women. *International Journal of Obesity*.

[99] Sugden JA, Davies JI, Witham MD, Morris AD, Struthers AD (2008). Vitamin D improves endothelial function in patients with Type 2 diabetes mellitus and low vitamin D levels. *Diabetic Medicine*.

[100] Jorde R, Figenschau Y (2009). Supplementation with cholecalciferol does not improve glycaemic control in diabetic subjects with normal serum 25-hydroxyvitamin D levels. *European Journal of Nutrition*.

[101] Zittermann A, Frisch S, Berthold HK (2009). Vitamin D supplementation enhances the beneficial effects of weight loss on cardiovascular disease risk markers. *American Journal of Clinical Nutrition*.

[102] von Hurst PR, Stonehouse W, Coad J (2010). Vitamin D supplementation reduces insulin resistance in South Asian women living in New Zealand who are insulin resistant and vitamin D deficient-a randomised, placebo-controlled trial. *British Journal of Nutrition*.

[103] de Boer IH, Tinker LF, Connelly S (2008). Calcium plus vitamin D supplementation and the risk of incident diabetes in the women’s health initiative. *Diabetes Care*.

[104] Oh J, Weng S, Felton SK (2009). 1,25(OH)_2_ vitamin D inhibits foam cell formation and suppresses macrophage cholesterol uptake in patients with type 2 diabetes mellitus. *Circulation*.

[105] Joergensen C, Gall M-A, Schmedes A, Tarnow L, Parving H-H, Rossing P (2010). Vitamin D levels and mortality in type 2 diabetes. *Diabetes Care*.

[106] Weisberg SP, McCann D, Desai M, Rosenbaum M, Leibel RL, Ferrante AW (2003). Obesity is associated with macrophage accumulation in adipose tissue. *Journal of Clinical Investigation*.

[107] Tsui H, Paltser G, Chan Y, Dorfman R, Dosch HM (2011). ‘Sensing’ the link between type 1 and type 2 diabetes. *Diabetes/Metabolism Research and Reviews*.

[108] Bastard J-P, Maachi M, Lagathu C (2006). Recent advances in the relationship between obesity, inflammation, and insulin resistance. *European Cytokine Network*.

[109] Arner P (2005). Insulin resistance in type 2 diabetes—role of the adipokines. *Current Molecular Medicine*.

[110] Vettor R, Milan G, Rossato M, Federspil G (2005). Review article: adipocytokines and insulin resistance. *Alimentary Pharmacology and Therapeutics, Supplement*.

[111] Trayhurn PW, Wood IS (2004). Adipokines: inflammation and the pleiotropic role of white adipose tissue. *British Journal of Nutrition*.

[112] Gandhi H, Upaganlawar A, Balaraman R (2010). Adipocytokines: the pied pipers. *J Pharmacol Pharmacother*.

[113] Fantuzzi G (2005). Adipose tissue, adipokines, and inflammation. *Journal of Allergy and Clinical Immunology*.

[114] Sánchez-Muñoz F, García-Macedo R, Alarcón-Aguilar F, Cruz M (2005). Adipocinas, tejido adiposo y su relación con células del sistema inmune. *Gaceta Médica de México*.

[115] Bado A, Levasseur S, Attoub S (1998). The stomach is a source of leptin. *Nature*.

[116] Stehno-Bittel L (2008). Intricacies of fat. *Physical Therapy*.

[117] Fain JN, Madan AK, Hiler ML, Cheema P, Bahouth SW (2004). Comparison of the release of adipokines by adipose tissue, adipose tissue matrix, and adipocytes from visceral and subcutaneous abdominal adipose tissues of obese humans. *Endocrinology*.

[118] Procaccini C, Lourenço EV, Matarese G, La Cava A (2009). Leptin signaling: a key pathway in immune responses. *Current Signal Transduction Therapy*.

[119] Martín-Romero C, Santos-Alvarez J, Goberna R, Sánchez-Margalet V (2000). Human leptin enhances activation and proliferation of human circulating T lymphocytes. *Cellular Immunology*.

[120] Matarese G, Lechler RI (2004). Leptin in intestinal inflammation: good and bad gut feelings. *Gut*.

[121] de Rosa V, Procaccini C, Calì G (2007). A key role of leptin in the control of regulatory T Cell proliferation. *Immunity*.

[122] Santos-Alvarez J, Goberna R, Sánchez-Margalet V (1999). Human leptin stimulates proliferation and activation of human circulating monocytes. *Cellular Immunology*.

[123] Sennello JAF, Fayad R, Pini ME (2008). Interleukin-18, together with interleukin-12, induces severe acute pancreatitis in obese but not in nonobese leptin-deficient mice. *Proceedings of the National Academy of Sciences of the United States of America*.

[124] Löfgren P, Andersson I, Adolfsson B (2005). Long-term prospective and controlled studies demonstrate adipose tissue hypercellularity and relative leptin deficiency in the postobese state. *Journal of Clinical Endocrinology and Metabolism*.

[125] Natali GLE, Pedro M, Fernando B Adiponestina: una adipocitoquina con múltiples funciones protectoras. *Acta Bioquíminca Clínica*.

[126] Pérez-Mayorga M (2007). El adipocito como órgano endócrino. Implicaciones fisilogicas y terapeuticas. *Revue Médicale*.

[127] Moreno MJ, Martínez JA (2002). Tejido adiposo: órgano de almacenamiento y órgano secretor. *Anales Sis San Navarra*.

[128] Steppan CM, Brown EJ, Wright CM (2001). A family of tissue-specific resistin-like molecules. *Proceedings of the National Academy of Sciences of the United States of America*.

[129] Yamauchi T, Kamon J, Waki H (2001). The fat-derived hormone adiponectin reverses insulin resistance associated with both lipoatrophy and obesity. *Nature Medicine*.

[130] Arner P (2005). Resistin: yet another adipokine tells us that men are not mice. *Diabetologia*.

[131] Steppan CM, Bailey ST, Bhat S (2001). The hormone resistin links obesity to diabetes. *Nature*.

[132] Milan G, Granzotto M, Scarda A (2002). Resistin and adiponectin expression in visceral fat of obese rats: effect of weight loss. *Obesity Research*.

[133] Rajala MW, Obici S, Scherer PE, Rossetti L (2003). Adipose-derived resistin and gut-derived resistin-like molecule-*β* selectively impair insulin action on glucose production. *Journal of Clinical Investigation*.

[134] Kusminski CM, McTernan PG, Kumar S (2005). Role of resistin in obesity, insulin resistance and Type II diabetes. *Clinical Science*.

[135] Hug C, Lodish HF (2005). Visfatin: a new adipokine. *Science*.

[136] Jia SH, Li Y, Parodo J (2004). Pre-B cell colony-enhancing factor inhibits neutrophil apoptosis in experimental inflammation and clinical sepsis. *Journal of Clinical Investigation*.

[137] Ognjanovic S, Bryant-Greenwood GD (2002). Pre-B-cell colony-enhancing factor, a novel cytokine of human fetal membranes. *American Journal of Obstetrics and Gynecology*.

[138] Chen M-P, Chung F-M, Chang D-M (2006). Elevated plasma level of visfatin/pre-B cell colony-enhancing factor in patients with type 2 diabetes mellitus. *Journal of Clinical Endocrinology and Metabolism*.

[139] Fukuhara A, Matsuda M, Nishizawa M (2005). Visfatin: a protein secreted by visceral fat that Mimics the effects of insulin. *Science*.

[140] Mackay CR (2001). Chemokines: immunology’s high impact factors. *Nature Immunology*.

[141] Zlotnik A, Yoshie O (2000). Chemokines: a new classification system and their role in immunity. *Immunity*.

[142] Rossi D, Zlotnik A (2000). The biology of chemokines and their receptors. *Annual Review of Immunology*.

[143] Liles WC, van Voorhis WC (1995). Review: nomenclature and biologic significance of cytokines involved in inflammation and the host immune response. *Journal of Infectious Diseases*.

[144] Recasens M, Ricart W, Fernández Real J (2004). obesidad e inflamación. *Revista de medicina de la Universidad de Navarra*.

[145] Ruan H, Hacohen N, Golub TR, Van Parijs L, Lodish HF (2002). Tumor necrosis factor-*α* suppresses adipocyte-specific genes and activates expression of preadipocyte genes in 3T3-L1 adipocytes: nuclear factor-*κ*B activation by TNF-*α* is obligatory. *Diabetes*.

[146] Böni-Schnetzler M, Boller S, Debray S (2009). Free fatty acids induce a proinflammatory response in islets via the abundantly expressed interleukin-1 receptor I. *Endocrinology*.

[147] Marcano Y, Torcat J, Ayala L, Verdi B, Lairet C, Maldonado M (2006). Funciones endocrinonas del tejido adiposo. Revisión. *Revista Venezolana de Endocrinología y Metabolismo*.

[148] Aguilar-Salinas CA (2007). Adiposidad Abdominal como factor de riesgo para enfermedades Crónicas. *Salud Pública de México*.

[149] Fontana L, Eagon JC, Trujillo ME, Scherer PE, Klein S (2007). Visceral fat adipokine secretion is associated with systemic inflammation in obese humans. *Diabetes*.

[150] Maedler K, Sergeev P, Ehses JA (2004). Leptin modulates *β* cell expression of IL-1 receptor antagonist and release of IL-1*β* in human islets. *Proceedings of the National Academy of Sciences of the United States of America*.

[151] Maedler K, Størling J, Sturis J (2004). Glucose- and interleukin-1*β*-induced *β*-cell apoptosis requires Ca^2+^ influx and extracellular signal-regulated kinase (ERK) 1/2 activation and is prevented by a sulfonylurea receptor 1/inwardly rectifying K^+^ channel 6.2 (SUR/Kir6.2) selective potassium channel opener in human islet. *Diabetes*.

[152] Stienstra RJ, Koenen L, van der Meer T, Tack CJ (2010). The inflammasome-mediated caspase-1 activation controls apipocyte differentiation and insulin sensitivity. *Cytokine*.

[153] Toda Y, Tsukada J, Misago M, Kominato Y, Auron PE, Tanaka Y (2002). Autocrine induction of the human pro-IL-1*β* gene promoter by IL-1*β* in monocytes. *Journal of Immunology*.

